# Labour market attachment among parents and self-rated health of their offspring: an intergenerational study

**DOI:** 10.1093/eurpub/ckz213

**Published:** 2019-12-03

**Authors:** Louise Lindholdt, Thomas Lund, Johan H Andersen, Merete Labriola

**Affiliations:** 1 Department of Public Health, Aarhus University, Aarhus,Denmark; 2 Research Centre for Youth and Employment, Regional Hospital West Jutland, University Research Clinic, Herning, Denmark; 3 Centre for Social Medicine, Frederiksberg and Bispebjerg Hospital, Copenhagen, Denmark; 4 Department of Public Health, University of Copenhagen, Copenhagen, Denmark; 5 Department of Occupational Medicine, Danish Ramazzini Centre, University Research Clinic, Regional Hospital West Jutland, Herning, Denmark; 6 NORCE, Norwegian Research Centre, Bergen, Norway

## Abstract

**Background:**

Unemployment influences the individual’s health, whether this effect passes through generations is less studied. The aim of this intergenerational study was to investigate whether parents’ labour market attachment (LMA) were associated with self-rated health (SRH) among adolescents using preceding labour market events.

**Methods:**

The study was performed using questionnaire data from the Danish Future Occupation of Children and Adolescents cohort (the FOCA cohort) of 13 100 adolescents (mean age 15.8 years) and their accompanying parents identified through registers. Adolescents’ SRH was measured using one item from SF-36. Information on parents’ LMA was obtained from a national register, analyzed on a weekly basis in a 5-year period before the adolescents completed the questionnaire. An integration indicator was calculated from an initial sequence analysis to determine how well the parents were integrated in the labour market. The association between the adolescents’ SRH and parents’ LMA was examined by logistic regression and an extended sequence analysis stratified on adolescents’ SRH.

**Results:**

Totally, 29.1% of the adolescents reported moderate SRH. The adjusted odds ratios (OR) of moderate SRH was higher among adolescents of parents with low labour market integration (OR: 1.5 95% CI: 1.3–1.6 for fathers and OR: 1.4 95% CI: 1.2–1.5 for mothers). Also, adolescents with moderate SRH had parents who were less integrated in the labour market and had more weeks on non-employment benefits compared with the adolescents, who reported high SRH.

**Conclusions:**

Unstable LMA among parents affected SRH among their adolescent children, indicating a negative effect of labour market marginalization across generations.

## Introduction

Key developmental periods in life are of specific importance to people’s health. Focussing on life course experiences can contribute to understanding exposures, which are influencing health in these periods and later in life.^1^

An important life course experience is working life.^1^ Being without work, incapacitated, long-term work absent or unemployed can impair health and well-being.^2^^,^^3^

Although the effects of not having a labour market attachment (LMA) have been found to go beyond the affected individual and have adverse effects on their family,^4^^,^^5^ sparse research has been done on how unemployment and unstable LMA among parents may influence their offspring’s health.

The literature unambiguously identifies parental employment status as a risk factor for impaired health among adolescents on various measures of health, including negative influence on health and well-being,^4^^,^^6^^,^^7^ poorer self-esteem,^8^ psychosomatic problems and chronic illness.^9^ Additionally, negative life-style changes caused by poor LMA has been found to be associated with low self-rated health (SRH),^10^ which is a strong predictor of morbidity and mortality.^11^^,^^12^

However, previous studies have addressed limitations in the research, stemming from the data and methods used to analyze the impact of parental LMA on adolescents’ health.^4^^,^^13^ Most of these studies were cross-sectional studies, using self-reported data on LMA, where the definitions of employment status were often assessed by point estimates, providing no knowledge on pre- and proceeding labour market events or the specific nature of transitions that affect the children’s health.

Over the last decade, the increase in quality of longitudinal data on nationally representative samples has expanded life-course epidemiology into public health research and has added to the understanding of the individual trajectories over time, but the pathways and mechanisms across generations remain much less clear.^14^ By combining quality longitudinal register data and survey data, the aim of this intergenerational study was to investigate whether parents’ LMA were associated with SRH among adolescents using preceding labour market events.

## Methods

### Data

Data were derived from the Future Occupation of Children and Adolescents Cohort (the FOCA cohort), a Danish longitudinal nationwide youth cohort, inviting all eligible adolescents attending the 9th grade (the graduating year from compulsory school).^15^ The FOCA cohort comprised 13 100 adolescents, consisting of 6685 girls (51.0%), and 6415 boys (49.0%) with an average age of 15.9 years. The adolescents represented all possible school types in the Danish school system and were widely distributed across Denmark. Data were collected in the first quarter of 2017, where the adolescents were recruited primarily through their schools. All attending adolescents answered a comprehensive online questionnaire.^15^^,^^16^

The data from the FOCA cohort were linked to the adolescents’ unique personal identification number given to all Danish citizens at birth.^17^ This enabled an accurate linkage to the adolescents’ parents through The Fertility Database of Statistics Denmark,^18^ which contained information on relations between children and their parents based on administrative registers. This linkage allowed studying the effects of factors across generations using the adolescents’ self-reported questionnaires and data from registers on their parents.

### Study population

**Figure 1:**
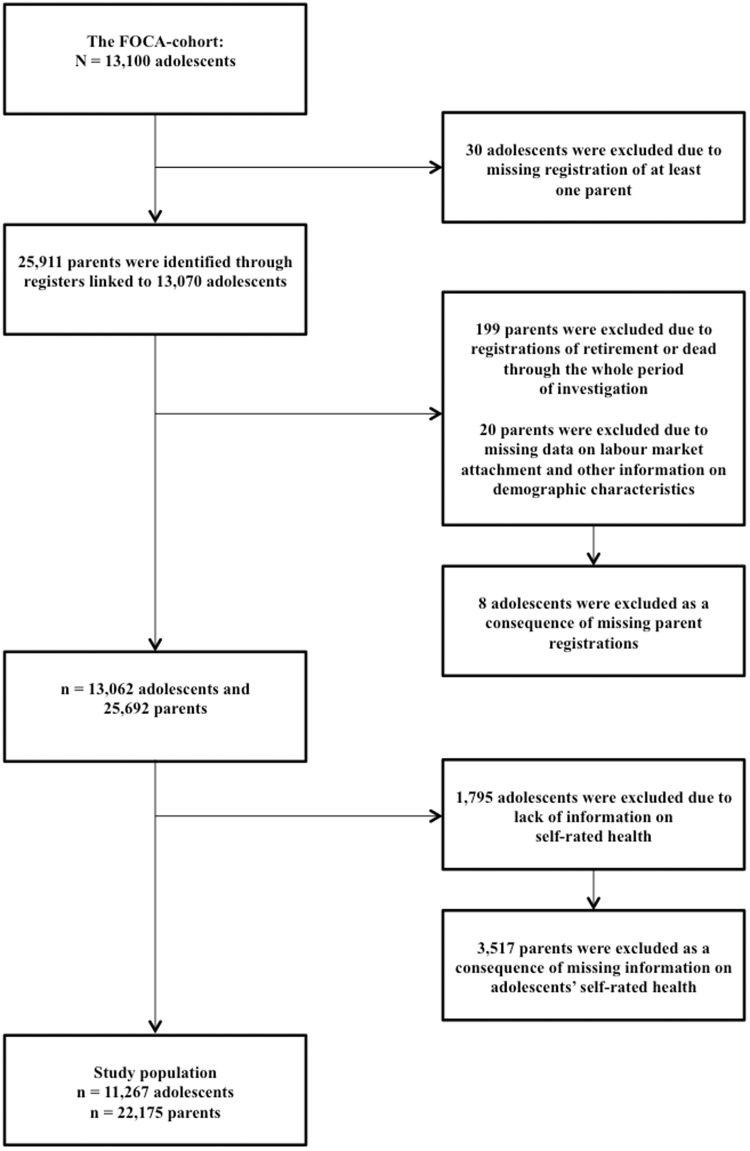
Flow of participants

A total of 25 911 parents were identified and linked to 13 070 adolescents (figure 1).

All adolescents linked to at least one parent, who was not retired or dead through the full period of investigation were included. Parents with missing information on LMA and other significant information on demographic characteristics were excluded. This resulted in a linkage of 25 692 parents to 13 062 adolescents.

A further inclusion criterion was complete information on main variables, thus 1795 adolescents were excluded due to lack of information on SRH, with a corresponding exclusion of their respective parents. This yielded a final study population of 11 267 adolescents, 5905 girls (52.4%) and 5362 boys (47.6%) with an average age of 15.8 years. The distribution of parents per adolescent was as follows: 10 908 adolescents were linked to two parents and 359 adolescents were linked to one parent, thus equalling 22 175 parents (table 1). Moreover, 21 862 parents were unique, which meant that some parents were linked to more than one adolescent.

**Table 1:** Characteristics of the study population

	n (%) or mean (SD)
**Adolescents**	11 267
Sex (%)	
Boys	5362 (47.6)
Girls	5905 (52.4)
Age, mean (years) (SD)	15.8 (0.4)
General self-rated health (%)	
High	7988 (70.9)
Moderate	3279 (29.1)
Family type (%)	
One-parent family	359 (3.2)
Two-parent family	10 908 (96.8)
**Parents** ^a^	21 862
Father (%)	10 815 (49.5)
Mother (%)	11 047 (50.5)
Age, mean (years) (SD)	47.4 (5.2)
Educational level (%)	
<10 years	3116 (14.6)
10–12 years	10 115 (47.2)
13–15 years	5926 (27.7)
>15 years	2256 (10.5)
**Parents’ LMA within 5 years** ^b^	
Average number of weeks in, mean (SD)	
Employment	209.5 (86.2)
Active relief	21.6 (51.9)
Passive relief	8.7 (39.9)
Sickness absence	8.3 (20.6)
Permanent out of the labour market	10.6 (49.7)
Emigration	2.2 (19.7)
Average number of episodes in, mean (SD)	
Employment	2.3 (3.2)
Active relief	1.3 (3.1)
Passive relief	0.1 (0.7)
Sickness absence	1.0 (3.0)
Permanent out of the labour market	0.1 (0.2)
Emigration	0.0 (0.2)
Average number of episodes (total), mean (SD)	4.8 (7.7)
Average number of different elements (total), mean (SD)	1.7 (0.8)
Volatility indicator, mean (SD)	82.8 (26.8)
Integration indicator, mean (SD)	80.6 (33.6)

^a^The amount of unique parents.

^b^Retrospectively from the time the adolescents completed the questionnaire.

### Measures

#### Self-rated health

The adolescents’ SRH was measured by the one item question from the Short-Form Health Survey (SF-36) assessing their general health as ‘excellent, very good, good, fair or poor’.^19^^,^^20^ In accordance with categorizations used in previous studies,^13^^,^^21^ SRH was dichotomized for the purpose of the statistical analyses, where ‘excellent’ and ‘very good’ health were categorized as high SRH and ‘good’, ‘fair’ and ‘poor’ health were categorized as moderate SRH.

#### Parents' labour market attachment

Information on parents’ LMA was obtained from the Danish Register for Evaluation of Marginalization (DREAM).^22^^,^^23^ DREAM contains national administrative data on employment and specific types of social transfer payments on all Danish citizens. The information was registered on a weekly basis, and if no information was registered, the individual was considered to be employed or self-supported.^22^

The parents of the adolescents were followed in DREAM for 5 years retrospectively from the time the adolescents completed the questionnaire. This period reflected the parents’ labour market trajectory. Based on the weekly registrations, six categories of labour market status were made: (i) employment, (ii) active relief, (iii) passive relief, (iv) sickness absence, (v) permanent out of the labour market and (vi) emigration. Employment was defined as being employed or self-employed in the labour market on ordinary terms. Active relief as being unemployed and receive social benefits indicating employability, receiving of the Danish students’ Grants or maternity pay that can lead to a positive working life course progression. Contrary, passive relief defined unemployment and receiving social benefits indicating no employability and a state far from the labour market. Sickness absence contained long-term sickness absence and part-time sickness absence. Permanent out of the labour market was defined as a withdrawal from the labour market either due to retirement pension or disability pension. Also, death was included in this group.

Two indicators expressing LMA were calculated based on the six categories.

A volatility indicator captured the quality of possible transitions between the different labour market states calculated as the proportion of episodes in employment and active relief relative to the total number of episodes.^24^ Transitions from a negative labour market state to employment or active relief reflect a positive transition, as it either represent a transition into full employment or a positive step towards employment, and thus a potential, positive labour market trajectory. The volatility indicator ranged from 0 to 100, where higher order values represented higher quality of transitions.

An integration indicator was calculated to determine how well the parents were integrated in the labour market. The quality of labour market integration was assessed as the sum of episodes within employment, which were weighted by the position within each sequence, where longer or more episodes in employment indicated a higher quality of labour market integration.^24^ The integration indicator ranged from 0 to 100, where high values represented well-integrated parents.

Number of episodes, episode lengths, number of elements and the average duration of weeks of the elements were calculated. Elements indicated the parents’ specific state of LMA created on the six constructed categories, and episodes were defined as identical consecutive elements.

#### Parents’ highest completed education

Information on the parents’ educational level was collected from the Danish Education Register of Statistics Denmark,^25^ and classified into four categories based on the highest level of completed education: <10 years of education (compulsory school), 10–12 years of education (upper secondary education and vocational education), 13–15 years of education (short-cycle higher education, medium-cycle higher education and bachelor’s degree) and >15 years of education (long-cycle higher education, PhD and research education).^26^

### Statistical analysis

Three statistical analyses were performed:

- Descriptive statistics on characteristics of the adolescents and their parents were examined and an initial sequence analysis was made in order to analyze the parents’ successions of labour market states, showing a complete event history on a weekly basis within a 5-year period (table 1). Although data were not normally distributed, the results were presented as mean values as the estimates in the sequence analysis otherwise would be incomprehensible.
- The indicator of the parents’ labour market integration calculated from the sequence analysis was used in a separate logistic regression analyzing the association between parents’ LMA and adolescents’ SRH. The integration indicator was used as the independent variable, dichotomized as above/below mean, and SRH as the dependent variable. A crude analysis and a fully adjusted analysis presenting odds ratios with 95% confidence intervals were made, adjusted for the adolescents’ sex, number of parents (one or two parents) and parents’ highest completed education. The analyses were performed separately for mothers and fathers. As some parents had more than one child in the cohort, robust standard errors were used.
- In addition to the logistic regression, a sequence analysis was performed on the impact of parental LMA on adolescent’s SRH. The analysis was constructed as aggregated characteristics of the parents’ labour market sequences stratified on the adolescents’ SRH, by exploring the parents’ specific labour market states on a weekly basis in a 5-year period.

STATA statistical software v. 15.1 was used for all the statistical analyses.

### Ethics

The Danish Data Protection Agency and the Ethics Committee of Statistics Denmark approved the FOCA cohort, approval no. 1-16-02-461-16, which complies with national legislation for use of data for research. The questionnaire was anonymous and voluntary to answer, and there was an option to withdraw from participation in the study at any time. The treatment of data was strictly confidential.

## Results

A total of 3279 adolescents (29.1%) reported moderate SRH while 7988 adolescents (70.9%) reported high SRH. In average, the parents were 209.5 weeks (80.3%) in employment, 21.6 weeks (8.3%) in active relief and 8.7 weeks (3.3%) in passive relief during the 5 years of investigation totalling 261 weeks with reference to the initial sequence analysis (table 1).

Additional analysis among the parents of non-responders indicated that the corresponding figure was 199.9 weeks in employment (76.6%). For other parameters, non-responders did not differ from responders (analysis not shown).

The calculated indicator of the parents’ labour market integration was used in a separate logistic regression analyzing the association between parents’ LMA and adolescents’ SRH. Parents with a below mean level of LMA correspond to 25.1%. For both fathers and mothers with a below mean level of labour market integration, the most adjusted models, considering the adolescents’ sex, number of parents and parents’ educational level, yielded an excess risk of moderate SRH among their offspring (table 2).

**Table 2:** Odds ratios (95% CI) for the association between parents’ labour market integration and moderate SRH among adolescents

	Fathers		Mothers	
	Model 1	Model 2	Model 1	Model 2
	(*n* = 10 971)	(*n* = 10 739)	(*n* = 11 204)	(*n* = 10 984)
Labour market integration				
Under mean	1.6 (1.4–1.7)	1.5 (1.3–1.6)	1.5 (1.4–1.6)	1.4 (1.2–1.5)
Over mean	1.0	1.0	1.0	1.0
Sex				
Girls		1.4 (1.3–1.5)		1.4 (1.3–1.5)
Boys		1.0		1.0
Number of parents				
One parent		0.8 (0.4–1.6)		1.1 (0.9–1.5)
Two parents		1.0		1.0
Parental education				
<10 years		1.6 (1.4–1.9)		1.9 (1.6–2.3)
10–12 years		1.3 (1.2–1.5)		1.4 (1.2–1.7)
13–15 years		1.0 (0.9–1.2)		1.3 (1.1–1.6)
>15 years		1.0		1.0

According to the extended sequence analysis, the adolescents who reported moderate SRH had parents who were less employed compared with adolescents who reported high SRH (table 3). A mean difference of 17.0 weeks within employment emerged during the 5 years of investigation in favour of the parents to the adolescents who reported high SRH. Contrary, adolescents who reported moderate SRH had parents who had more weeks on social transfer payments or were outside the labour market (table 3).

**Table 3:** Aggregated characteristics of parents’ labour market sequences stratified on adolescents’ SRH

	High SRH		Moderate SRH		
	Mean (SD)	Min/max	Mean (SD)	Min/max	Diff.
Average number of weeks in					
Employment	214.5 (82.6)	0; 261	197.5 (93.0)	0; 261	17.0
Active relief	19.8 (50.1)	0; 261	25.9 (55.6)	0; 261	−6.1
Passive relief	7.4 (36.8)	0; 261	12.1 (46.5)	0; 261	−4.7
Sickness absence	7.7 (19.6)	0; 234	9.8 (22.7)	0; 240	−2.1
Permanent out of the labour market	9.7 (47.4)	0; 261	12.9 (54.6)	0; 261	−3.2
Emigration	2.0 (18.7)	0; 261	2.9 (21.8)	0; 261	−0.9
Average number of episodes in					
Employment	2.3 (3.1)	0; 56	2.5 (3.4)	0; 53	−0.2
Active relief	1.2 (3.1)	0; 65	1.5 (3.2)	0; 45	−0.3
Passive relief	0.1 (0.6)	0; 17	0.2 (0.7)	0; 11	−0.1
Sickness absence	0.9 (3.0)	0; 64	1.1 (3.1)	0; 52	−0.2
Permanent out of the labour market	0.0 (0.2)	0; 4	0.1 (0.3)	0; 3	−0.1
Emigration	0.0 (0.1)	0; 3	0.0 (0.2)	0; 3	0.0
Average number of episodes (total)	4.6 (7.6)	1; 129	5.4 (8.1)	1; 105	−0.8
Average number of different elements in sequence	1.7 (0.8)	1; 5	1.8 (0.9)	1; 5	−0.1
Volatility indicator	84.1 (25.9)	0; 100	79.6 (28.5)	0; 100	4.5
Integration indicator	82.5 (32.2)	0; 100	75.9 (36.4)	0; 100	6.6

Both the integration indicator and the volatility indicator showed that adolescents who reported moderate SRH had parents who were less integrated in the labour market (mean integration indicator of 75.9) and had a little poorer quality of transitions within the different labour market states (mean volatility indicator of 79.6) compared with the parents of the adolescents who reported high SRH (mean integration indicator of 82.5 and mean volatility indicator of 84.1) (table 3).

## Discussion

Parental LMA in a 5-year period prior to baseline assessment of SRH was associated with their adolescent children’s health in multiple ways. The adolescents with moderate SRH (approximately 30% of the sample) more often had parents with less employment and more time on social transfer payments in the preceding 5-year period. Besides, these parents had more frequent changes of employment status, indicating a more unstable employment pattern. This trend was confirmed by analyzing a construct reflecting labour market integration among the parents, where an integration score below the mean was associated with moderate SRH among the adolescents. After adjusting for adolescents’ sex, number of parents and parents’ educational level, this association was only modestly attenuated. However, a comparison of parental LMA between adolescent responders and non-responders showed differences in the average time in employment where parents of non-responders tended to have fewer weeks in employment. This difference could potentially cause an underestimation of the association between parents’ LMA and their adolescent children’s health. Therefore, the risk estimates provided in this study should be considered as conservative.

The finding of parental LMA as a risk factor for moderate SRH status among adolescent health is in line with previous findings. Sleskova et al.^4^ found that parental unemployment was negatively associated with subjective health of their adolescent children when considering the duration of unemployment. Especially, the father’s employment status was a predictor of moderate SRH.

Reinhardt Pedersen and Madsen^9^ found that children and adolescents of parents without employment had higher prevalence of recurrent psychosomatic symptoms and low well-being compared with children where at least one parent was employed during a 6-month period. Another study by Bacikova-Sleskova et al.^21^ found a negative association between the father’s employment status on several health indicators including SRH among their adolescent children, but no effect from the mother’s employment status.

Despite differences in the way of measuring parental LMA, a negative impact of unemployment among parents on their offspring’s health is found in this study as well as other previous studies.

In this study, the parents’ trajectories of LMA were investigated in a 5-year period prior to when the adolescents completed the questionnaire. This time span was chosen for two equally important reasons. The 5-year period covered the beginning of adolescence, defined by WHO^27^ as the developing life period between 10 and 19 years. As changes and impacts in this critical life period have proven to have consequences for health both during adolescence but also through the life-course,^28^ this period was chosen to assess whether the adolescents’ SRH were affected by changes stemming from their parents’ preceding labour market trajectories. Additionally, the time span should obtain a comprehensive overview of the parents’ labour market trajectories rather than just a cross section of the actual labour market status at the time the adolescents completed the questionnaire. By applying a time dimension using sequence analysis with 5 years of investigation, it was possible to clarify the dynamics of the parents’ labour market trajectories, which would not have been possible using a point estimate of LMA. Labour market trajectories are diverse and often complex, and due to its exploratory method, sequence analysis is capable of handling the complexity and multiple outcomes of the parents’ labour market trajectories.

The effect of one parent’s LMA on the adolescents’ SRH was captured and not the combined effect of both parents. This can be considered a weakness in the sense that it does not reflect the situation in a household perspective, given that there is no knowledge of what it means for the adolescents’ SRH if one parent has a high quality of LMA and the other parent has a low. Conversely, it can be seen as a strength as it hereby addresses the impact of either the mother’s or the father’s degree of LMA on the child.

The use of data from the Danish registers is a strength of this study. Both the Danish Education Register and DREAM are considered to be of high quality with low risk of misclassification bias and less non-missing information through follow-up.^23^^,^^25^ Lack of detailed information on employment status has previously been considered a limitation when studying parental LMA.^4^ Therefore, this study used information from the DREAM register to create categories of the parents’ LMA, as it contains specific labour market states on a micro level based on employment data and detailed information on social transfer payments, which enables capturing the dynamics of the parents’ labour market trajectories.

Since DREAM is particularly useful in longitudinal analyses, it was obvious to use this data to generate a more nuanced picture of parental labour market trajectories, which has not previously been used in studies of associations between parents’ employment patterns and their children’s health.

Based on the Danish regulations and policies of employment, the six categories were constructed in order to provide a certain level of detail in examining LMA by including specific possibilities of labour market states reflecting both positive and negative progressions in the working life course. However, as the aim of this study was merely to determine whether parents’ LMA had an association with adolescent children’s health, the aggregation of information into six labour market categories was a pragmatic choice and constructed specifically for this study.

Adolescents’ SRH is based on self-reported data originating from the one-item question on general health from SF-36, which is incorporated in the FOCA cohort questionnaire. Even though the use of self-reported data can lead to inaccuracies, the validity of this item seems acceptable, as the measure used is validated for people of 14 years and older.^19–20^ The item was dichotomized in high SRH including the response categories ‘excellent’ and ‘very good’ and moderate SRH including ‘good’, ‘fair’ and ‘poor’. Additionally, the analyses were tested with another dichotomization of SRH to see possible differences in the results. This caused changes in the risk estimates, but the trend remained the same although the group capturing moderate SRH was very small.

In conclusion, the study finds that not being in work can have negative consequences beyond the individual. In a public health perspective, it is therefore not merely of importance to support the individual so the time spent on social transfer payments becomes as short as possible, but equally important to consider the negative health effects across generations. In order to understand this, it would be interesting to build even more on the current analysis by examining the specific pathways within the labour market trajectories on a micro level of parents of adolescents with moderate SRH.

## Funding

The study is part of the PUSAM project funded by The Danish Working Environment Research Fund (Project no.24-2013-09).

*Conflicts of interest*: None declared.

**Key points**

- Adolescents with moderate SRH, more often had parents with less employment and more time on social transfer payments, than adolescents with high SRH.
- An unstable LMA and a low degree of integration in work over time among parents, was associated with low SRH among their adolescent children.
- The study underlines the importance of recognizing how marginalization from the labour market can have detrimental health effects over long periods of time and across generations.
